# Hemoglobin consumption by *P. falciparum* in individual erythrocytes imaged via quantitative phase spectroscopy

**DOI:** 10.1038/srep24461

**Published:** 2016-04-18

**Authors:** Matthew T. Rinehart, Han Sang Park, Katelyn A. Walzer, Jen-Tsan Ashley Chi, Adam Wax

**Affiliations:** 1 Dept. of Biomedical Engineering, Duke University, Durham, NC 27708, US; 2 Department of Molecular Genetics and Microbiology, Duke University School of Medicine, Durham, NC 27710, US.

## Abstract

*Plasmodium falciparum* infection causes structural and biochemical changes in red blood cells (RBCs). To quantify these changes, we apply a novel optical technique, quantitative phase spectroscopy (QPS) to characterize individual red blood cells (RBCs) during the intraerythrocytic life cycle of *P. falciparum*. QPS captures hyperspectral holograms of individual RBCs to measure spectroscopic changes across the visible wavelength range (475–700 nm), providing complex information, i.e. amplitude and phase, about the light field which has interacted with the cell. The complex field provides complimentary information on hemoglobin content and cell mass, which are both found to dramatically change upon infection by *P. falciparum*. Hb content progressively decreases with parasite life cycle, with an average 72.2% reduction observed for RBCs infected by schizont-stage *P. falciparum* compared to uninfected cells. Infection also resulted in a 33.1% reduction in RBC’s optical volume, a measure of the cells’ non-aqueous components. Notably, optical volume is only partially correlated with hemoglobin content, suggesting that changes in other dry mass components such as parasite mass may also be assessed using this technique. The unique ability of QPS to discriminate individual healthy and infected cells using spectroscopic changes indicates that the approach can be used to detect disease.

*P. falciparum* is the primary cause of malaria, which is responsible for over 500,000 deaths each year world-wide^1^. Early diagnosis is essential for appropriate treatment, and many of these deaths occur in countries that lack the resources to screen every suspected case of malaria. The current gold standard of malaria screening requires a highly trained microscopist to prepare and evaluate stained slides; however, staining reagents and trained technicians are not always available in many high-risk locations. As an alternative, rapid diagnostic tests (RDTs) are inexpensive, convenient, and do not require highly-skilled microscopists. However they suffer from reduced diagnostic accuracy and provide no information on parasite staging^2^. In developed countries, PCR-based diagnosis has become the standard as it is highly sensitive. On the other hand, PCR-based detection requires costly infrastructure and reagents to perform, preventing its widespread use in resource poor settings^3^,^4^,^5^,^6^,^7^. Thus, there is a strong need for new diagnostic techniques that combine the advantages of skilled microscopy (sensitivity, specificity, and single-cell staging capabilities) with the convenience, automation, and cost-effectiveness of RDTs.

Quantitative phase microscopy (QPM) techniques have been advanced to make label-free quantitative morphology measurements at the single-cell level. QPM has previously been applied to the study of *P. falciparum* biophysics extensively over the past decade^8^,^9^,^10^,^11^,^12^,^13^,^14^. Extensions of QPM include tomographic phase microscopy (TPM)^9^,^14^ and optical diffraction tomography (ODT)^13^ which have been used to map the 3D refractive index (RI) of infected RBCs at progressive parasite infection stages. QPM of dynamic samples has been used to map temporal membrane fluctuations of RBCs, demonstrating differences in shear modulus across various parasite stages^14^, therapeutic treatments^9^, and genetic variants^10^. One research group has even performed a proof-of-concept study to use holographic phase images to develop an automated algorithm capable of distinguishing individual infected cells from uninfected cells^8^. However, these studies all rely on literature values of RI determined at a particular, single wavlength to derive RBC metrics, which may not accurately determine Hb content. For accurate assessment of cell contents, a measurement method that can uniquely identify and quantify specific molecular components is needed.

Although QPM is highly sensitive to microscopic morphology and refractive index changes, the approach lacks molecular specificity, an important feature in many diagnostic approaches. During the *Plasmodium spp.* life cycle, up to 80% of the intracellular hemoglobin is consumed and converted into inert hemozoin crystals^15^. Thus, accurate quantification of decreases in hemoglobin content or increases in hemozoin content could be valuable in providing a parasite-specific signature as well as determining stage of infection. TPM and ODT estimate hemoglobin content by assuming that the intracellular RI is composed exclusively of an aqueous hemoglobin solution and therefore are unable to distinguish between RI changes due to hemoglobin versus other proteins that may contribute to dry mass^9^,^13^,^14^. Other recent efforts to measure specific biochemical changes in single *Plasmodium-*infected RBCs include the combination of Raman spectroscopy with QPM^12^ or performing single-cell infrared imaging spectroscopy^16^.

In recent years, QPM has been expanded to provide molecular specificity by incorporating hyperspectral imaging to measure spatially-resolved phase dispersion across the visible wavelength range. Quantitative phase spectroscopy (QPS) uses measurements of phase changes as a function of wavelength to identify specific molecules. This is a distinct difference from absorbance measurements where changes in the amplitude of transmitted light as a function of wavelength reveals molecular information. Instead, spectral variation in the phase of transmitted light, i.e., phase dispersion, provides knowledge of the complex refractive index which can also be used to provide molecular information.

The first demonstration of QPS quantified hemoglobin concentration in aqueous samples at millimolar concentrations^17^; further efforts applied the approach to examine hemoglobin content of an individual RBC^18^. Oxy-hemoglobin is a good target for QPS because it exhibits two strong absorption peaks in the visible wavelength range and offers a large molar extinction coefficient. As predicted by the Kramers-Kronig relations^19^, the absorption properties of Hb produce features in the complex refractive index which can be revealed with QPS measurements, even though other molecules with absorption signatures in the visible spectrum may not be apparent. Hemoglobin content is usually quantified as an average mass across a population of red blood cells, the mean corpuscular hemoglobin (MCH). However, the ability of QPS to quantify hemoglobin in microscopic quantities enables application of the technique to individual RBCs, possibly offering new diagnostic capabilities for detecting rare infected cells. While examples of hemoglobin measurements in individual RBC’s have been seen in the literature^18^,^20^, these typically only show a few isolated cells and do not examine changes due to pathology or disease.

In this paper, we seek to incorporate molecular specificity into holographic characterization of individual RBCs by applying QPS to analyze the optical spectra of RBCs at various stages of *P. falciparum* infection. Spectra from individual RBC at ring, trophozoite and schizont stages are analyzed to determine their hemoglobin content (in pg) and compared to those for uninfected cells. A progressive decrease in hemoglobin is observed with infection stage with some variability from cell to cell. This result is compared to changes in optical volume of the cell to confirm that changes in Hb content are not due to merely size variations. The ability to distinguish Hb content from other changes in cell dry mass content provide a unique capability for QPS which can be leveraged for better characterization of *P. falciparum* infection using holographic imaging. The accuracy and limitations of this approach are discussed and compared to previous measurements with an eye towards diagnostic capacity.

## Methods

### Quantitative phase spectroscopy scheme

**Figure 1:**
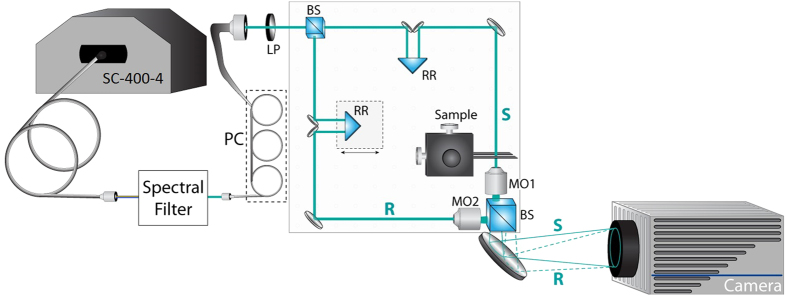
Quantitative Phase Spectroscopy system: (PC) polarization controller, (LP) linear polarizer, (BS) beam splitter, (RR) retroreflector mirror pair, (MO) microscope objective. Sample (S) and reference (R) beams are imaged by matched MOs onto the camera.

The quantitative phase spectroscopy (QPS) instrument has been described previously^17^ and is presented in Fig. 1. A supercontinuum laser (Fianium SC-400-4) spanning 450–750 nm is used for illumination. The broadband light is filtered to produce a 1.12 nm spectral full-width at half-maximum (FWHM) bandwidth with a tunable center wavelength using a spectral filter. The filter disperses light using a 300 lp/mm transmission diffraction grating (Thorlabs, GT25-03), and a galvanometric scanning mirror (GSM) to pass only a selected bandwidth as input to the interferometer. The filtered light is passed through a single-mode fiber (Thorlabs, 405-XP) to remove the spatio-spectral variation, and a polarization controller paddle (PC) and subsequent linear polarizer (LP) are used to minimize polarization variations. After this conditioning, approximately 150 μW of optical power is delivered into the interferometer as a collimated beam.

The interferometer splits the incident light into reference (R) and sample (S) arms, which are path-matched using mirror-based retroreflectors (RR) mounted on translation stages. The pathlength of the arms is adjusted when a sample is introduced into the system in order to maximize interferometric efficiency. Because the illumination light has a finite bandwidth of 1.12 nm, the arms must be matched within the coherence length (83–193 μm, depending on center wavelength) in order to produce interference. As discussed previously, a narrower illumination line width relaxes the constraint of path-matching^17^. The sample is imaged onto the camera using MO1 (Zeiss Plan-NeoFLUAR 40 × 0.75NA), producing an effective magnification of ~107×. The reference beam is imaged onto the camera using a matched microscope objective (MO2), and an off-axis fringe is produced by adjusting the lateral position of the reference arm and MO2.

Off-axis interferograms are captured by a high-speed CMOS camera (Photron FastCam SA-4, 1024 × 1024 px, 10-bit data capture) capable of acquiring 3600 frames per second (fps) at full resolution. As the tunable wavelength filter is stepped across a wavelength range of 475–700 nm in 5 nm increments, the camera is externally-triggered to acquire a burst of four off-axis interferograms at each wavelength with an integration time of 1 millisecond per image. In order to minimize spectral artifacts due to low-speed drift between the two interferometer arms, eight spectral sweeps are sequentially captured, producing a total of 32 off-axis interferograms at each wavelength over a time period of 5 seconds.

The processing of off-axis interferograms has been described previously^17^,^21^. Briefly, each interferogram is spatially filtered in the Fourier (spatial frequency) domain around the carrier frequency associated with the off-axis fringe. The filtered information is re-centered in Fourier space and inverse Fourier transformed to yield a two-dimensional complex image of the wavefront differences between the sample and reference arms of the interferometer. The complex holographic amplitude and phase images at each wavelength are averaged across repeated exposures of the sample to reduce noise. A reference set of hyperspectral amplitude- and phase-images with no cells in the field of view are captured separately for each physical sample imaged to enable removal of phase and amplitude artifacts arising from non-uniformities in illumination.

As demonstrated previously, holographic images must be digitally focused to the correct axial plane in order to preserve quantitative accuracy when computing single-cell metrics^22^. Digital refocusing was performed on a spectrally-averaged sample hologram of each field of view to determine the plane of best-focus for quantitative analysis.

### Single cell spectral analysis

Both the amplitude and phase of the transmitted field can be used to determine the total mass of hemoglobin within each cell. Changes in amplitude as a function of wavelength can be used to determine the absorbance of each cell, *A*_*cell*_. Absorbance is the exponential attenuation factor describing the change in amplitude. It is calculated as:


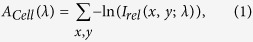


where *I*_*rel*_ (*x*, *y*;*λ*) is the measured intensity image, defined to be the square of the amplitude. In general, absorbance is related to the decadic molar extinction coefficient *ε*_*HbO2*_ (*λ*) by:


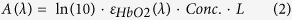


where *L* is the pathlength through the absorber and concentration is expressed in terms of moles/volume. In the experiments presented below, the pathlength of the absorbers are not known. Thus to extract concentration of Hb from absorbance, pathelength would be needed. However, the product of concentration and pathlength yields a quantity with units of moles/area. Therefore, integrating *A*_*cell*_ over the cell’s projected surface area yields the total molar quantity of hemoglobin which is easily converted to hemoglobin mass using its molar mass constant.

The spectral phase images also can be analyzed to produce a measurement of hemoglobin mass. To implement this calculation, we use a measurement of the cells’ solid components which we have termed optical volume (OV)^22^:


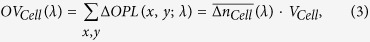


where Δ*OPL*(*x*, *y*; *λ*) = Δ*φ*(*x*, *y*; *λ*)/2*π* are the measured relative optical path delays of the light through the sample. OV is equal to the product of 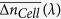, the average RI of the cell, and *V*_*cell*_ the physical volume of the cell. However, these two parameters cannot be measured independently with our approach. Instead, the OV metric is measured as a function of wavelength, to provide a parameter that represents the cells solid components. In cell analysis, dry mass is usually recovered from phase measurements using a similar approach but it depends on knowledge of the refractive index increment of the material *α* = Δ*n*/*Conc.*^23^,^24^,^25^. Due to the distinctive spectral features of hemoglobin, fitting the OV spectrum yields a measurement of hemoglobin mass. First, the OV volume is fit to a three-term Cauchy equation, 
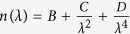
 to account for volumetric contributions arising from RI mismatch and dispersion. The residual spectral features are attributed to hemoglobin and can provide a mass measurement by least squares fitting of the relative RI increment of hemoglobin, Δ*α*_*HbO2*_(*λ*). We note that this problem is simplified due to the strong absorptive features of Hb but if other molecules with significant absorptive features were present, a multivariate analysis could be used to simultaneously determine their concentrations.

### Imaging and Analysis Procedures

RBCs were infected with *P. falciparum* and synchronized using methods described by Saliba and Jacobs-Lorena^26^ During the 48 hour life cycle, centrifugation in a Percoll density gradient was used to separate uninfected RBCs from infected RBCs containing parasites. Briefly, infected red cells are centrifuged at room temperature for 5 minutes at 1000 rpm (201 × g). Supernatant from pelleted culture is removed, resuspended at 50% hematocrit and placed atop a Percoll gradient comprised of 3 mL of 70% Percoll-sorbitol at the base and 3 mL of 40% Percoll-sorbitol solution on top. The resuspended culture and gradient are then centrifuged at 3,500 rpm (2,300 × g) for 20 minutes at room temperature.

**Figure 2:**
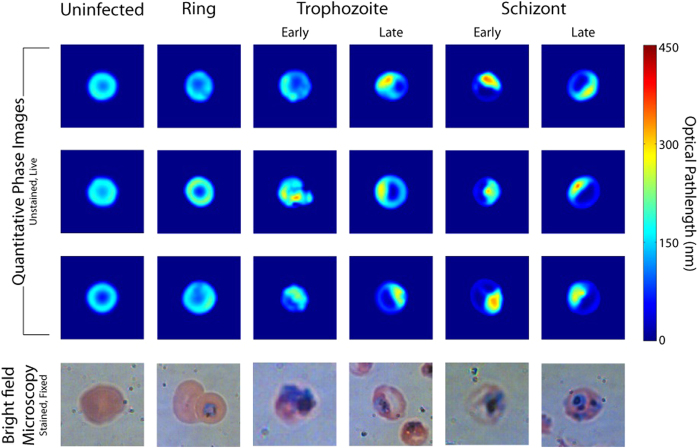
Representative quantitative phase images of RBCs in each stage, as well as brightfield microscopy of stained cells. Phase images have been spectrally-averaged across the visible range (475–700 nm).

Cells were isolated and imaged at multiple time-points, corresponding to the morphological stages identified by pathologists: ring (10 hrs), early trophozoite (22 hrs), late trophozoite (34 hrs), early Schizont (42 hrs) and late Schizont (46 hrs). Using the QPS system, unlabeled RBCs at each stage were imaged in an aqueous environment (99:1 Dulbecco’s phosphate buffered saline, D8662 Sigma-Aldrich, to bovine albumin fraction V (7.5%), 15260–037 Gibco) and a corresponding histologic slide was made by fixing & staining the cells (Fig. 2). Each field of view acquired with the QPS system contained between 5 and 20 individual cells. RBCs were segmented semi-automatically by: (1) applying a phase threshold of Δ*ϕ* >0.2  *radians* to identify all potential-RBC objects in the field of view, (2) exclusion of objects larger or smaller than certain threshold sizes as either cell clumps or non-RBC objects, (3) verification by a human analyst that all individual RBCs were included and that no clumps of cells had been included for analysis. Individual cell morphology was not considered when isolating and screening cells.

RBCs whose OV spectra contained phase wrapping artifacts were automatically discarded from further analysis. The remaining cell morphology images were inspected by three independent observers, two trained in QPM image analysis and one trained in histological analysis of malaria infection. Each observer identified each RBC as either infected or uninfected. Only cells that were identified unanimously as infected were used for analysis of the infected groups.

## Results

### Measured hemoglobin loss from infected RBCs

**Figure 3:**
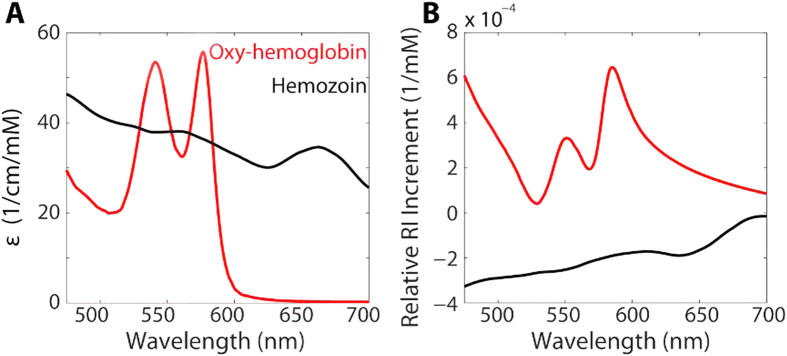
(**A**) Molar extinction coefficients of oxy-hemoglobin^28^ and *P. falciparum* by-product hemozoin^27^,^29^. (**B**) Corresponding relative RI increments calculated via the Kramers-Kronig relations^19^.

*P. falciparum* grows within RBCs and consumes hemoglobin as a fuel source. The consumption of hemoglobin produces free heme that would otherwise be toxic both to the parasite and the RBC. To mitigate this toxicity, *P. falciparum* creates insoluble heme dimer crystals called hemozoin. In addition to the morphological changes of infected RBCs, this process reduces the total amount of hemoglobin within a cell and creates the hemozoin byproduct with its own distinct spectral features^27^. Figure 3 presents the molar extinction coefficients of oxy-hemoglobin^28^ and hemozoin^27^,^29^ and the respective corresponding relative RI increments, as calculated by the subtractive Kramers-Kronig relations referenced to *λ*_0_ = 800 *nm*^19^.

RBCs contain a very high concentration of oxy-hemoglobin (32–36 g/dL or 5.0–5.6 mM reference range). When hemoglobin is converted to hemozoin, the double-peak features in the 500–600 nm spectral range almost disappear, and a small absorption peak appears at 650–665 nm^27^. Hemozoin also lacks the large absorption feature in the Soret band (~400 nm) which hemoglobin exhibits, and therefore possesses a relative RI increment that *increases* with wavelength when calculated from the molar extinction spectrum over the range of 200–900 nm.

**Figure 4:**
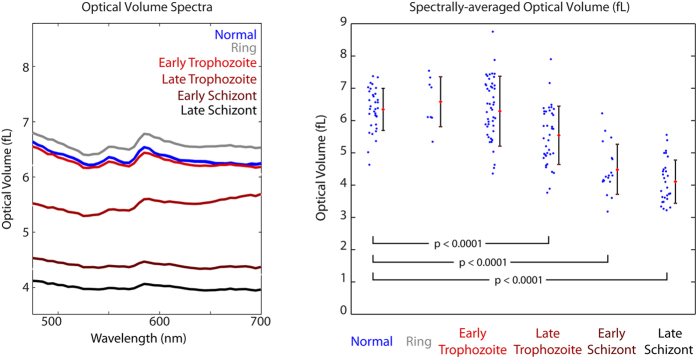
(**a**) Stage-separated OV spectra. (**b**) Spectrally-averaged OV across cell groups examined in this study. Cells infected by ring-stage and early trophozoite parasites show no significant OV changes. Late Trophozoite, Early Schizont, and Late Schizont infected RBCs show a progressive decrease in OV with highly statistically significant differences compared to uninfected and ring stage.

As noted above, both the amplitude and phase of the transmitted light field can be used to determine the total mass of hemoglobin within each cell. The OV spectrum is calculated from the phase of transmitted light for each cell and the average for each population is shown in Fig. 4 (a). The spectrally-averaged OV of uninfected RBCs was found to be significantly higher than that of the infected cells (Fig. 4(b)), even though RBC’s infected with *P. falciparum* may exhibit a larger *maximum* OPL, as seen in Fig. 2. Cells infected with *P. falciparum* also display a wider range of volumes, potentially indicating variation in the dynamics of parasite growth and hemoglobin consumption. Table 1 summarizes the OV measurements for each of the populations. Significant decreases in OV were seen for all infection stages except for the ring and early trophozoite stages. In addition to differences in the averaged OV, Fig. 4(a) also shows that the nonlinear spectral features associated with oxy-hemoglobin (520–600 nm) are more prominent in the uninfected RBC population.

**Figure 5:**
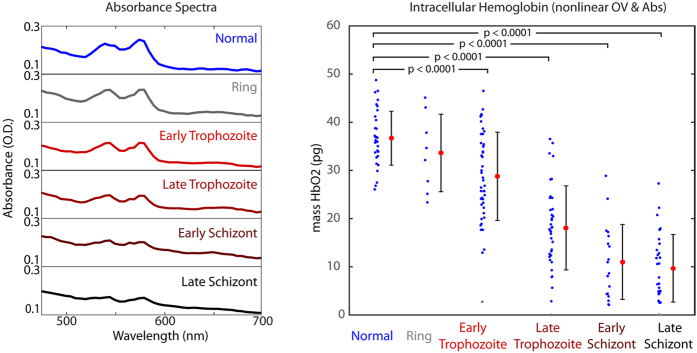
(**a**) Stage-separated absorbance spectra. (**b**) Individual cell hemoglobin mass, calculated by least-squares fitting of the hemoglobin-specific absorptive and refractive spectral features (combined average of OV and absorbance measurements for each cell). All stages except for Ring stage show significant changes in intracellular hemoglobin (p < 0.0001 for all significance t-tests).

The amplitude of transmitted light can also provide a measurement of Hb content. Analysis of the absorbance spectra for the two populations reveals a decrease in the average quantity of hemoglobin present (Fig. 5). The overall absorbance increases with infection, however the characteristic hemoglobin peaks diminish significantly. The absorbance spectra are fit using non-negative linear least-squares regression to the form *A*(*λ*) = *C*_1_+ −*C*_2_· *λ* + *C*_3_ *ε*_*HbO*2_ (*λ*) to recover mass-density concentration maps, which are then integrated over the cell area to estimate the mass of hemoglobin contained within each cell. Only oxyHb is included in this fit since deoxyHb and metHb were found to not be substantial contributors to the spectra (~5% for deoxyHb, 0% for metHb) and thus were omitted from the analysis.

OV spectra are fit to the form 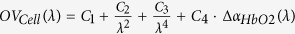 using linear least-squares regression with a non-negative constraint on the fourth term to recover the mass of hemoglobin within each cell. Again deoxyHb and metHb were found to not be substantial components and were omitted in the presented analysis. Table 2 presents Hb concentration data averaged over the indicated RBC populations for each of the two methods (OV and Absorbance) as well as the average of the two measurements, which was shown previously to improve the precision of concentration determination^30^. To simplify the analysis, two additional groups are also presented as aggregates, early and late trophozoite and early and late schizont. Fig. 5(b) graphically shows the computed Hb mass for each stage of infection examined in this study. All of the disease stages except for the ring-stage cells show highly significant changes in Hb content when compared to the uninfected population of RBCs using a two-sided unpaired t-test.

The Hb concentration measurements show a distinct difference between uninfected cells, measured to contain 36.7 ± 5.6 pg (mean ± standard deviation) of oxy-hemoglobin, and infected cells, found to contain as little as 9.7 ± 7.0 pg (late schizont stage). Thisis a decrease in Hb of 27.0 pg, or 73.6% (p < 0.0001, t-test, JMP Pro). We note that the quantity of oxyHb measured in uninfected cells with this approach is somewhat higher than laboratory reference ranges for mean cell Hb (29 ± 2 pg^31^, measured across >10^6^ cells). To determine the influence of these systematic differences, several uninfected RBC samples were measured with a hematology analyzer (Sysmex KX-21N) to determine hemoglobin content and the results compared to the QPS measurements. The analysis showed that the response was linear with a 0.823 proportionality constant and a small offset (0.14 pg). Using this factor to adjust the QPS measurements, uninfected cells were found to have 30.1 ± 4.6 pg, in line with reference values, and infected late schizont cells were found to have 7.8 ± 5.8 pg. The percentage decrease remains constant with this adjustment but its absolute magnitude is reduced to 22.3 pg.

**Figure 6:**
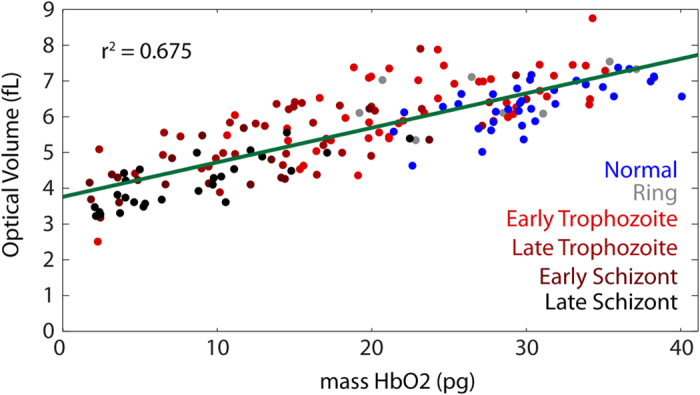
Relationship between non-spectroscopic OV and mass of hemoglobin within individual cells. Both infected and uninfected cells show only a weak trend between these metrics.

As a final analysis, the relationship between the total optical volume for a cell, i.e. the average OV across the visible spectrum, and the fitted mass of hemoglobin of each cell is examined (Fig. 6). It appears that for both uninfected and infected cells hemoglobin content is only a modest predictor of cell optical volume (coefficient of determination r^2^ = 0.675). Thus, combining both hemoglobin mass and averaged OV could improve the ability to discriminate between these cell types as compared to using a single metric alone.

## Discussion

Malaria is a leading cause of death worldwide, with *P. falciparum* infection being the primary causative agent. Detection of infection, determination of parasitemia (extent of infection), and estimation of the stages of parasites within their lifecycles are critical for effective diagnosis and subsequent treatment. The need for better understanding of *P. falciparum’s* lifecycle and improved diagnostic techniques has motivated significant attention amongst quantitative phase imaging researchers. QPM has previously been applied to the investigation of RBCs infected by *P. falciparum* to estimate cell and parasite volumes, RIs, and mechanical properties^9^,^13^,^14^. However, spectroscopic changes in individual infected RBCs have not previously been investigated by QPM. In this study, QPS has been shown to measure statistically significant decreases in both RBC optical volume and Hb mass associated with parasite infection. The measured decrease in hemoglobin mass of schizont-stage infected RBCs relative to healthy RBCs (72.2%) is larger than literature reports of hemoglobin consumption by *P. falciparum* (50–55%)^27^, but there are no data available for single cell measurements, rather just aggregate measures.

In this study, two different approaches are used to determine Hb mass. First, amplitude based attenuation is used to determine Hb mass from absorbance measurements. Second, phase measurements are used to determine the refractive index of the cell across the visible spectrum. The changes in RI can be analyzed to determine Hb mass but also quantify the other components of the cell which contribute to RI but do not cause absorbance changes. The results presented here show that changes in total OV and hemoglobin mass (Fig. 6) are only loosely correlated, suggesting that RI measurements at a single wavelength do not necessarily describe only hemoglobin concentration but also include other cell components. By using spectral information it is possible to separate the contribution of Hb to the RI, providing a more reliable method than RI measurements at a single wavelength alone^30^.

The loose correlation between OV and Hb mass illustrates the limitation of using single wavelength measurements to assess Hb content in QPM. Previous holographic phase microscopy studies of RBCs infected by *P. falciparum* have used single wavelength measurements to determine the 3-dimensional refractive index (RI)^14^. Although the primary goal of this earlier study was to understand the changes in membrane dynamics by observing and analyzing its fluctuations, measurements of Hb concentration and mass for a small number of RBCs were also presented. The average RI from their measurement was compared to literature values to estimate Hb concentration which was then multiplied by the measured cell volume to obtain total Hb mass. The obtained values are generally in agreement with literature values for uninfected cells but significantly lower than those reported here for trophozoite- and schizont-stage infected cells. While the small number of cells studied and the variability observed in Hb consumption by the parasite can account for this difference, there were several assumptions made in converting the tomography measurements into hemoglobin estimates. The most significant assumption was that the entirety of the non-aqueous RI contributions of the cell was due to Hb. However, the results presented above show that upon analyzing the full spectrum for individual RBCs only a portion of the cell contents can be attributed to Hb. The second assumption is that the refractive index can be inverted to give hemoglobin concentration based on the literature values given in^32^. Unfortunately, there is no consensus value of the refractive index increment of Hb in the literature; rather, the reported values of *α*_*HbO2*_ at a fixed wavelength (λ = 589 nm) range from 0.148–0.2686 ml/g^32^,^33^,^34^,^35^. Thus the selection of a fixed-wavelength RI increment based on a literature value is somewhat arbitrary and raises questions about both the methodology and the accuracy of this measurement. In contrast, we have presented more useful and accurate measurements here based upon fitting wide-range spectral data with well-characterized Hb absorbance and relative RI increment spectra, and further comparing the resulting Hb measurements with those of a traditional hematology analyzer.

The uncorrected Hb mass measured here for uninfected RBCs (36.7 pg) is higher than reference ranges for mean cell hemoglobin (27–31 pg)^31^. Our direct comparison indicates a systematic increase of 17.7% in the QPS Hb measurements compared to the results of the hematology analyzer. It is reasonable that the analyzer would show a lower value, since it obtains an MCH measurement by first counting all of the cells using an impedance based measurement and then assessing the total Hb using an extinction measurement. Any cells or other cellular debris which did not contain Hb could add to the counted number but not the total Hb, resulting in a lower measurement on a per cell basis. Any variations in extinction measurement could also skew these results, with unaccounted-for effects such as multiple scattering also pushing the MCH value lower. On the other hand, the results presented here are based on fitting a curve with an expected spectral shape which can also be a source of error. However, given the low residuals and good fits seen in these results, this effect alone is not a likely cause of the discrepancy. Further analysis which compares these two approaches is needed.

The Hb mass and OV measurements both show statistically significant differences between uninfected and infected RBCs, however neither of these parameters nor a linear combination of the two fully differentiates the populations. When seeking to identify the stage of a single cell, the sensitivity improves with parasite stage (values based on a specificity of 91.2%), with poor discrimination seen for ring stage versus uninfected (37.5% sensitivity) and excellent discrimination for comparing schizont stage to uninfected (95.7% sensitivity). Discrimination of trophozoite stages was variable with good sensitivity for late trophozoite (89.2% sensitivity) but modest sensitivity for early trophozoite (62.5% sensitivity) identification. Overall, a linear combination of the Hb mass and OV measurements discriminates uninfected cells across all stages examined here with 77.1% sensitivity and 91.2% specificity. However, only a small number of ring stage infected cells (n = 8) were examined in this study. The range of sensitivity seen here is in part due to the variability of individual RBC volume, mass, and RI, and also in part due to the variability of metabolic activity and life cycle stage among individual parasites. The observed differences in these populations do, however, motivate further work to develop models that incorporate spectral features for characterizing and classifying the parasitic invasion of RBCs by *P. falciparum.*

## Conclusion

A pilot study has been presented which quantifies the spectral changes in individual RBCs infected by *P. falciparum* using QPS. Phase and amplitude spectra were processed to extract absorbance and optical volume spectra for populations of uninfected and infected cells. These spectra were then analyzed to estimate Hb content and mass differences across the populations. Both Hb mass and OV were seen to decrease significantly with infection, but were only loosely correlated with each other. This study indicates that spectral analysis can be useful for measuring the quantity of hemoglobin consumed by *P. falciparum* on a cell-by-cell basis, and can be useful in distinguishing infection and later parasite stages based on the degree of these changes.

## Additional Information

**How to cite this article**: Rinehart, M. T. *et al.* Hemoglobin consumption by *P. falciparum* in individual erythrocytes imaged via quantitative phase spectroscopy. *Sci. Rep.* **6**, 24461; doi: 10.1038/srep24461 (2016).

## Figures and Tables

**Table 1:** Spectrally-averaged OV of RBCs vs. stage.

Stage	#Cells	Optical Volume (OV)		Diff. vs. Uninfected		Significance
		Mean (fL)	St. dev (fL)	(fL)	(%)	(p-value)
Uninfected	34	6.35	±0.65	–	–	
Ring	8	6.58	±0.77	+0.23	+3.6%	0.3893
E. Troph	48	6.29	±1.08	−0.06	−0.9%	0.7735
L. Troph	37	5.54	±0.90	−0.81	−12.8%	<0.0001
Troph (Combined)	85	5.97	±1.07	−0.38	−6.0%	0.0202
E. Schiz	18	4.49	±0.78	−1.86	−29.3%	<0.0001
L. Schiz	29	4.11	±0.67	−2.24	−35.5%	<0.0001
Schiz (Combined)	47	4.25	±0.73	−2.10	−33.1%	<0.0001

**Table 2:** Change in individual cell hemoglobin in picograms (pg) during *P. falciparum* life cycle.

Stage	#Cells	Abs. via Amplitude		OV via Phase		Combined		Diff. vs. Uninf.		Significance
		Mean	Std	Mean	Std	Mean	Std	(pg)	(%)	(p-value)
Uninfected	34	37.43	±5.99	35.97	±7.94	36.70	±5.59	–	–	–
Ring	8	28.92	±6.69	38.35	±12.36	33.63	±8.05	−3.07	−8.4%	0.2000
E. Troph	48	25.88	±10.00	31.67	±9.97	28.78	±9.16	−7.92	−21.6%	<0.0001
L. Troph	37	14.32	±8.06	21.83	±10.81	18.07	±8.72	−18.63	−50.8%	<0.0001
Troph (Combined)	85	20.85	±10.82	27.39	±11.39	24.12	±10.39	−12.58	−34.3%	<0.0001
E. Schiz	18	9.58	±7.95	12.42	±9.41	11.00	±7.77	−25.70	−70.0%	<0.0001
L. Schiz	29	7.12	±7.60	12.26	±9.28	9.69	±7.01	−27.01	−73.6%	<0.0001
Schiz (Combined)	47	8.06	±7.75	18.75	11.21	10.19	±7.26	−26.51	−72.2%	<0.0001
